# Disulfiram Attenuates Osteoclast Differentiation *In Vitro*: A Potential Antiresorptive Agent

**DOI:** 10.1371/journal.pone.0125696

**Published:** 2015-04-30

**Authors:** Hua Ying, An Qin, Tak S. Cheng, Nathan J. Pavlos, Sarah Rea, Kerong Dai, Ming H. Zheng

**Affiliations:** 1 Centre for Orthopaedic Research, School of Surgery, The University of Western Australia, Perth, Australia; 2 Department of Orthopaedics, The Ninth People’s Hospital, Shanghai Jiao Tong University School of Medicine, Shanghai, China; 3 Harry Perkins Institute of Medical Research, Perth, Australia; 4 Department of Endocrinology & Diabetes, Sir Charles Gairdner Hospital, Perth, Australia; Faculté de médecine de Nantes, FRANCE

## Abstract

Disulfiram (DSF), a cysteine modifying compound, has long been clinically employed for the treatment of alcohol addiction. Mechanistically, DSF acts as a modulator of MAPK and NF-κB pathways signaling pathways. While these pathways are crucial for osteoclast (OC) differentiation, the potential influence of DSF on OC formation and function has not been directly assessed. Here, we explore the pharmacological effects of DSF on OC differentiation, activity and the modulation of osteoclastogenic signaling cascades. We first analyzed cytotoxicity of DSF on bone marrow monocytes isolated from C57BL/6J mice. Upon the establishment of optimal dosage, we conducted osteoclastogenesis and bone resorption assays in the presence or absence of DSF treatment. Luciferase assays in RAW264.7 cells were used to examine the effects of DSF on major transcription factors activation. Western blot, reverse transcription polymerase chain reaction, intracellular acidification and proton influx assays were employed to further dissect the underlying mechanism. DSF treatment dose-dependently inhibited both mouse and human osteoclastogenesis, especially at early stages of differentiation. This inhibition correlated with a decrease in the expression of key osteoclastic marker genes including CtsK, TRAP, DC-STAMP and Atp6v0d2 as well as a reduction in bone resorption *in vitro*. Suppression of OC differentiation was found to be due, at least in part, to the blockade of several key receptor activators of nuclear factor kappa-B ligand (RANKL)-signaling pathways including ERK, NF-κB and NFATc1. On the other hand, DSF failed to suppress intracellular acidification and proton influx in mouse and human osteoclasts using acridine orange quenching and microsome-based proton transport assays. Our findings indicate that DSF attenuates OC differentiation via the collective suppression of several key RANKL-mediated signaling cascades, thus making it an attractive agent for the treatment of OC-mediated disorders.

## Introduction

Bone is a dynamic organ undergoing constant remodeling to ensure correct mineral homeostasis and to maintain proper mechanical strength [1]. Bone remodeling is balanced by the complementary yet opposing activities of two principal bone cells i.e. bone-resorbing osteoclasts (OC) and bone forming osteoblasts (OB) [2]. It is now widely recognized that OCs are the principal cells responsible for bone resorption [3]. These large multinucleated cells are formed by the fusion of mononuclear progenitors of the monocyte–macrophage lineage [4]. The commitment and fusion of OC progenitors is governed primarily by two crucial cytokines, namely macrophage colony stimulating factor (M-CSF) and receptor activator of nuclear factor-B ligand (RANKL) produced by bone resident OBs, osteocytes and marrow stromal cells [5, 6]. Upon binding to its cognate receptor RANK (expressed on the surface of OC progenitors), RANKL triggers the activation of several downstream nuclear transcription factors among which nuclear factor kappa-light-chain-enhancer of activated B cells (NF-κB), activator protein 1 (AP-1) and nuclear factor of activated T-cells, cytoplasmic 1 (NFATc1) are indispensable for OC differentiation [7]. During their RANKL-driven maturation, the OC plasma membrane is furnished with specialized molecular machinery such as vacuolar H^+^-type ATPases (V-ATPases) proton pumps and chloride channels which facilitates its unique bone-resorbing function. The macromolecular V-ATPases are enriched on the bone-apposed ruffled border membrane (the OCs resorptive apparatus) and serve to couple ATP hydrolysis and proton secretion [8, 9], a process essential for the dissolution of inorganic bone matrix and the activation of bone digesting enzymes such as Cathepsin K [10]. Moreover, certain V-ATPase subunits have also been shown to relate to OC formation [11, 12].

While physiological osteoclastic bone resorption is essential for the maintenance of human skeleton, enhanced OC formation and/or function result in excessive bone resorption, leading to either localized osteolysis or systemic bone loss, as occurs in osteoporosis and bone metastasis [13, 14]. To combat this, several antiresorptive agents including bisphosphonates, estrogens, selective estrogen receptor modulators, calcitonin and monoclonal antibodies such as denosumab have been developed over the past few decades. Despite these agents showing promising potential to alleviate bone loss, each is, however, not without its limitations [15, 16]. Therefore, the discovery of new antiresorptives remains an unmet challenge. With the strict regulatory requirements imposed on the development of new drugs for antiresorptive therapy, rebadging market approved agents for alternative applications may offer a safe and cost effective treatment avenue for osteoporosis.

Disulfiram (DSF), a cysteine modifying compound, has long been employed (since the 1920s) for the clinical treatment of chronic alcoholism [17, 18]. Moreover, extensive data determined that DSF modulates mitogen-activated protein kinase (MAPK) and NF-κB pathways, leading to apoptosis of various cancer cell lines [19, 20]. In addition, DSF has also been identified as a potential inhibitor of the yeast V-ATPase proton pump [21]. Considering that MAPK, NF-κB pathways and V-ATPases all play crucial roles at various stages of the OC formation-bone resorption cycle, DSF represents an attractive antiresorptive candidate. Here, we have examined the antiresorptive potential and molecular action of DSF on OC formation, bone resorption and acidification. Our findings indicate that DSF potently inhibits OC differentiation and function *in vitro* via attenuation of RANKL-induced NF-κB and NFATc1 activation but does not influence V-ATPase activity. DSF may therefore represent a previously overlooked class of antiresorptive therapy.

## Materials and Methods

### Reagents

DSF, bafilomycin A1 (Baf), Acridine Orange (AO), nigericin and vanilomycin were purchased from Sigma-Aldrich (St. Louis, MO, USA). Mouse M-CSF and GST-RANKL_160-318_ (rRANKL) recombinant proteins were expressed and purified in our laboratory as previously described [22]. Human M-CSF were purchased from (R&D Systems, Minneapolis, MN, USA). CellTiter 96 AQ_ueous_ One Solution Cell Proliferation Assay (MTS) was purchased from Promega (Madison, WI, USA). Luciferase substrate was purchased from Promega (Madison, WI, USA). The α-MEM fetal bovine serum (FBS) and antibiotics were purchased from Gibco Invitrogen (Carsbad, CA, USA). Hank’s Balanced Salt Solution (HBSS) were purchased from Thermo Fisher Scientific (Scoresby, VIC, Australia). Primary antibodies used include mouse monoclonal anti-β-actin (1/5000), mouse monoclonal anti-NFATc1 (1/1000) (DSHB University of Iowa, USA), rabbit polyclonal anti-c-Fos (1/500), mouse monoclonal anti-p-ERK1/2 (1/1000), rabbit polyclonal anti-IκBα (1/1000) (Santa Cruz, CA, USA), rabbit monoclonal anti-p65 (1/1000), rabbit monoclonal anti-p-p38 (1/1000), rabbit monoclonal anti-p38 (1/1000), rabbit monoclonal anti-p-JNK (1/1000), rabbit polyclonal anti-JNK (1/1000), rabbit monoclonal anti-p-Src (1/1000), mouse monoclonal anti-Src (1/1000), rabbit polyclonal anti-p-Akt (1/1000), rabbit monoclonal anti-Akt (1/1000) (Cell Signaling, MA, USA), rabbit polyclonal anti-ERK1/2 (1/1000) (Promega, Madison, WI, USA), rabbit polyclonal anti-procathepsin K (1/2000) (Abcam, Cambridge, UK), rabbit polyclonal anti-Atp6v0d2 (1/500) (produced by our laboratory). All antibodies were used at the concentrations indicated above.

### Cytotoxicity assay

Adherent M-CSF-dependent bone marrow monocytes (BMM) isolated from C57BL/6J mice were seeded onto a 96-well plate at density of 1×10^4^ cells per well and treated with various concentrations of DSF for 72 hrs or treated with 200 nM DSF for different periods. Cell proliferation was measured after incubation with MTS solutions [23] using a microplate reader (BIO-RAD, Model 680). IC_50_ (50% inhibitory concentration) was calculated using GraphPad Prism 6.

### In vitro osteoclastogenesis and bone resorption

Adherent M-CSF-dependent BMMs were isolated from C57BL/6J mice as previously described [24]. The use of laboratory animal in the current study was approved by the University of Western Australia Animal Ethics Committee and all animal work was performed in accordance to approved guidelines outlined by the committee. Briefly, BMMs were seeded onto a 96-well plate at density of 6×10^3^ cells per well and stimulated with 100 ng/ml rRANKL and 30 ng/ml M-CSF in the presence or absence of DSF (12.5 nM, 25 nM, 50 nM, and 100 nM) at 37°C in 5% CO_2_ for 5 days for the formation of multinucleated OCs. After 5 days culture, cells were fixed in 4% paraformaldehyde (PFA) and stained in solutions containing 50 mM acetate buffer, 30 mM sodium tartrate, 0.1 mg/ml naphthol AS-MX phosphate and 0.3 mg/ml Fast Red Violet LB for 30 min to show TRAP activity. TRAP positive cells which contained 3 or more nuclei were scored as mature OCs, and cell spread area were measured using NIS-Elements BR.

For bone resorption assays, BMMs were seeded on bovine bone discs at 6×10^3^ cells/well and stimulated with rRANKL and M-CSF in the presence or absence of DSF for indicated time, bone discs were fixed in 4% PFA and stained for TRAP activity. Cells were removed by brushing and resorption pits were visualized after staining with 1% toluidine blue solution. The percentage of bone surface area resorbed was quantified using NIS-Elements BR.

### Human osteoclast culture

Human monocytes were isolated from whole blood as previously described [25]. The isolated monocytes were seeded into 96-well plates at a density of 1x10^6^ cells/well. Cells were cultured in complete α-MEM containing 10 ng/ml human M-CSF and 100 ng/ml rRANKL, with the culture medium replaced at an interval of 3 days for a total of 10 days before fixing with 4% PFA and staining for TRACP activity.

### Luciferase Assay

To examine the effects of DSF on RANKL-induced NF-κB and NFATc1 activation, RAW264.7 cells were transiently transfected with luciferase reporter genes (p-NF-κB-TA-Luc, p-NFAT-TA-Luc, pTA-Luc (Clontech, Mercury Pathway Profiling System) or pGL3-promoter (Promega)) as previously described [26, 27]. Cells were seeded (1.5×10^5^/well) into 48-well plates. After attachment, cells were pretreated with DSF for 1h and then stimulated with RANKL (100 ng/ml) for an indicated period of time. Luciferase activities were measured in cell lysates using the Promega Luciferase Assay System according the manufacturer’s instructions.

### Western blot analysis

Total cellular proteins were extracted from cultured BMMs or OCs using RIPA lysis buffer (50 mM Tris pH 7.5, 150 mM NaCl, 1% Nonidet P-40, 0.1% SDS, 1% sodium deoxycholate) supplemented with Protease Inhibitor Cocktail (Roche). Lysates were cleared by centrifugation at 16,000 g at 4°C for 20 mins and supernatants containing proteins were collected. For immunoblotting, 30μg of extracted proteins diluted in SDS-sampling buffer was resolved by SDS-PAGE (10–15%) gels and then electroblotted onto nitrocellulose membranes (Hybond ECL, Amersham Life Science). Following transfer, membranes were blocked with 5% (w/v) skim milk in TBS-Tween (TBS; 0.05 M Tris, 0.15 M NaCl, pH 7.5 and 0.1% Tween-20) for 1 hr and then probed with primary antibodies diluted in 1% (w/v) skim milk powder in TBS-Tween at 4°C overnight. Membranes were washed and then incubated with HRP-conjugated secondary antibodies and antibody reactivity was detected by the Western Lightning Ultra Detection Kit (PerkinElmer, Waltham, MA, USA) using the FujiFilm LAS-3000 Gel Documentation System (FujiFilm, Tokyo, Japan) and its associated software.

### Semi-quantitative Reverse Transcription (RT)-PCR

Total cellular RNA was isolated from cultured cells using PureLink RNA Mini Kit (Invitrogen) in accordance with the manufacturer’s protocol. For RT-PCR, single-stranded cDNA was reverse transcribed from 500 ng total RNA using MLV-RT with oligo-dT primer. All PCR was carried out using 1 μl of each cDNA using the following cycling parameters 94°C, 40 sec; 58°C, 40 sec; and 72°C, 40 sec. Primer sequences and cycle numbers are summarized in Table 1.

**Table 1:** Primer sequences and cycle numbers for semi-quantitative RT-PCR.

	Forward primer	Reverse primer	Cycle number
CtsK	5’-CCAGTGGGAGCTATGGAAGA-3’	5’-AAGTGGTTCATGGCCAGTTC-3’	25
TRAP	5’-TCCTGGCTCAAAAAGCAGTT-3’	5’-ACATAGCCCACACCGTTCTC -3’	28
DC-STAMP	5’-ACCTTGTTTCTTGGAACCAGAC-3’	5’-TGTCATTCATATGAGCCTCCA-3’	28
Atp6v0d2	5’-CCTGGTTCGAGGATGCAA-3’	5’-GGTCTCACACTGCACTAGGTTG-3’	28
β-actin	5’-AGCCATGTACGTAGCCATCC-3’	5’-CTCTCAGCTGTGGTGGTGAA-3’	25

PCR samples were analyzed by agarose gel electrophoresis. The expression level of all genes were transformed to grey scale by ImageJ 1.46r and normalized to the expression level of the housekeeping gene (β-actin).

### Intracellular acidification by acridine orange staining

Intracellular acidification was determined by acridine orange (AO) fluorescence quenching method. BMM-derived OCs or human OCs treated with DSF or Baf for 12 hrs were incubated with 10 μg/ml AO for 15 mins at 37°C. Cells were washed twice with Hank’s Balanced Salt Solution (HBSS) and processed for fluorescent microscopy analysis on a NIKON Eclipse TE2000-S fluorescence microscope and associated software or fluorescence measured on FLUOStar Optima spectrophotometer (BMG LabTech) at excitation of 485 nm and emission of 520 nm.

### Proton transport assay

Microsomes were isolated from the mouse BMMs according to an established protocol [28]. BMMs were washed twice in 10 ml of homogenization buffer (20 mM HEPES-KOH, 1 mM EDTA, 2 mM dithiothreitol, 250 mM sucrose, pH 7.4, at 4°C) and collected by centrifugation at 1,000 g for 10 min. The pelleted cells were then resuspended in 1 ml of the above buffer and passed 10 times through a 27-gauge needle. The homogenate was centrifuged for 10 min at 7000 rpm at 4°C to pellet unbroken cells and mitochondria. The supernatant was centrifuged at 100,000 g for 60 min in a Beckman TLA110 rotor. The pellet was suspended in buffer A (l50 mM KCl, 20 mM HEPES-KOH, 2mM dithiothreitol, pH7.4), and glycerol was added to the suspension to 25% of the final volume. The microsomes were used for the proton influx assay. The protocol was modified from Sørensen MG *et al*. [29]. Briefly, microsomes were incubated in reaction buffer (5 mM HEPES, 150 mM KCl, 5 mM MgCl_2_; pH 7.4) containing 10μM AO and 1.25μM valinomycin and the inhibitors were added as described in the figure legends. The reaction was incubated at 37°C for 60 min. The reaction was initiated by addition of ATP at a final concentration of 1.5 mM to the wells and monitored by measuring the uptake of AO using excitation of 485 nm and emission of 520 nm. The fluorescence was read every 5 seconds and the reaction was stopped after 300 seconds with the addition of 1.5 μM nigericin. The initial rate (-∆F/∆t) was calculated from the slope generated during the first 60 seconds. To ensure that the compounds had no effect on the fluorescence, the plate was read for 30 seconds before addition of ATP to obtain a steady level.

### Statistical analysis

All data are expressed as mean ± SD and represent three independent experiments for each experimental condition. Statistical analysis was evaluated with unpaired Student’s t-test to analyze differences between groups. The graphs and plots were produced with GraphPad Prism 6.

## Results

### Disulfiram attenuates osteoclast differentiation but does not influence bone resorption function

**Fig 1:**
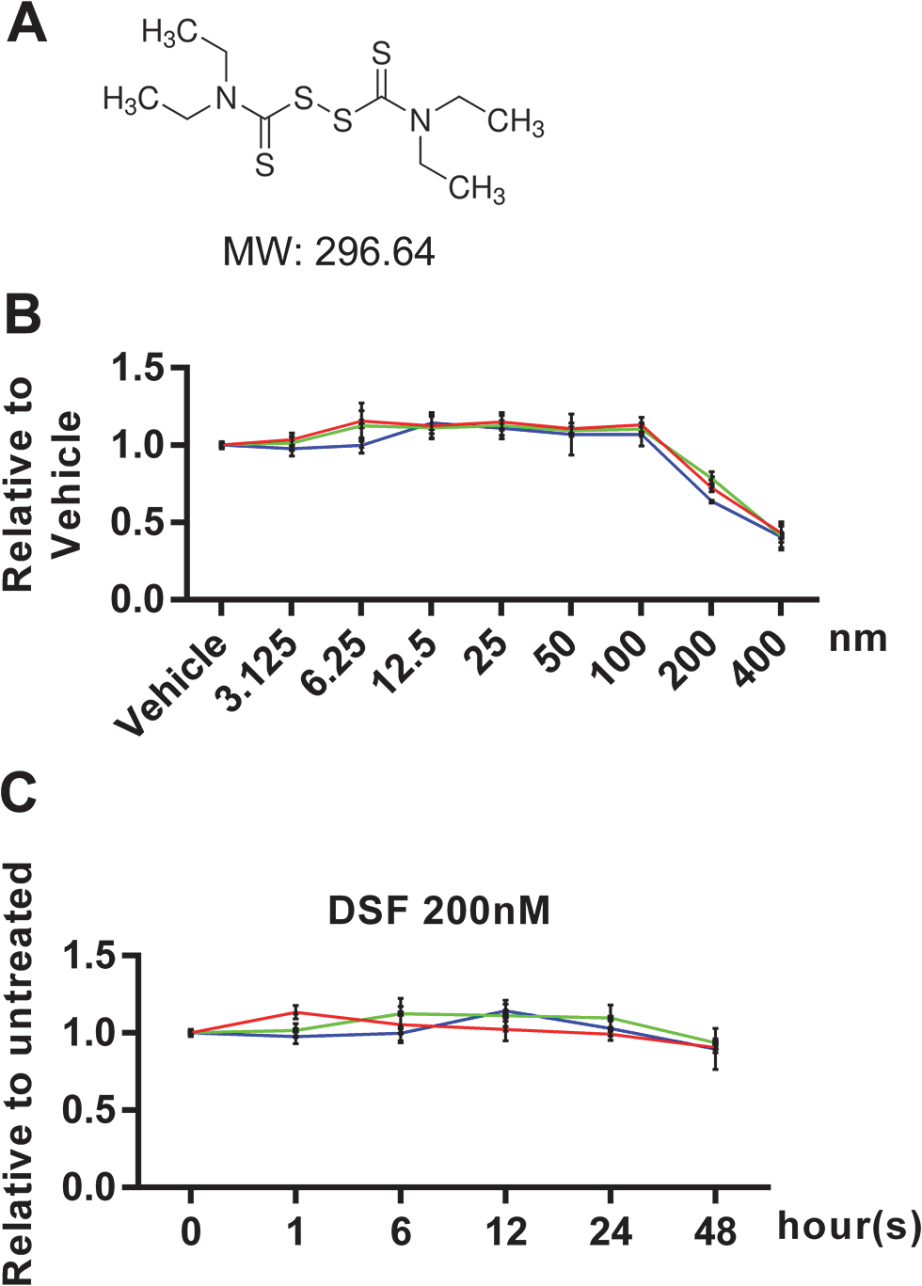
DSF-induced cytotoxicity in BMMs. (A) Chemical structure of DSF. (B and C) BMMs incubated with various concentrations of DSF for 72 hours or with 200 nM DSF for various time periods were stained with MTS assay solutions to determine the cytotoxicity of DSF. The calculated IC_50_ was 214.95 ± 14.78 nM (n = 3).

To test the pharmacological effect(s) of DSF (Fig 1A) on OC formation, we first established the half maximal inhibitory concentration (IC_50_) of DSF on OC progenitor cells (i.e. M-CSF-treated BMMs). To this end, OC progenitor cells were incubated with varying concentrations of DSF for up to 72 hours and assayed by MTS assay solutions, a measurement of cellular metabolic activity and cell viability. As shown in Fig 1B, the established IC_50_ of DSF was 214.95±14.78 nM (n = 3). Furthermore, OC progenitor cells were treated with 200 nM DSF for different time periods with no obvious cytotoxic effect up to 48 hours (Fig 1C).

**Fig 2:**
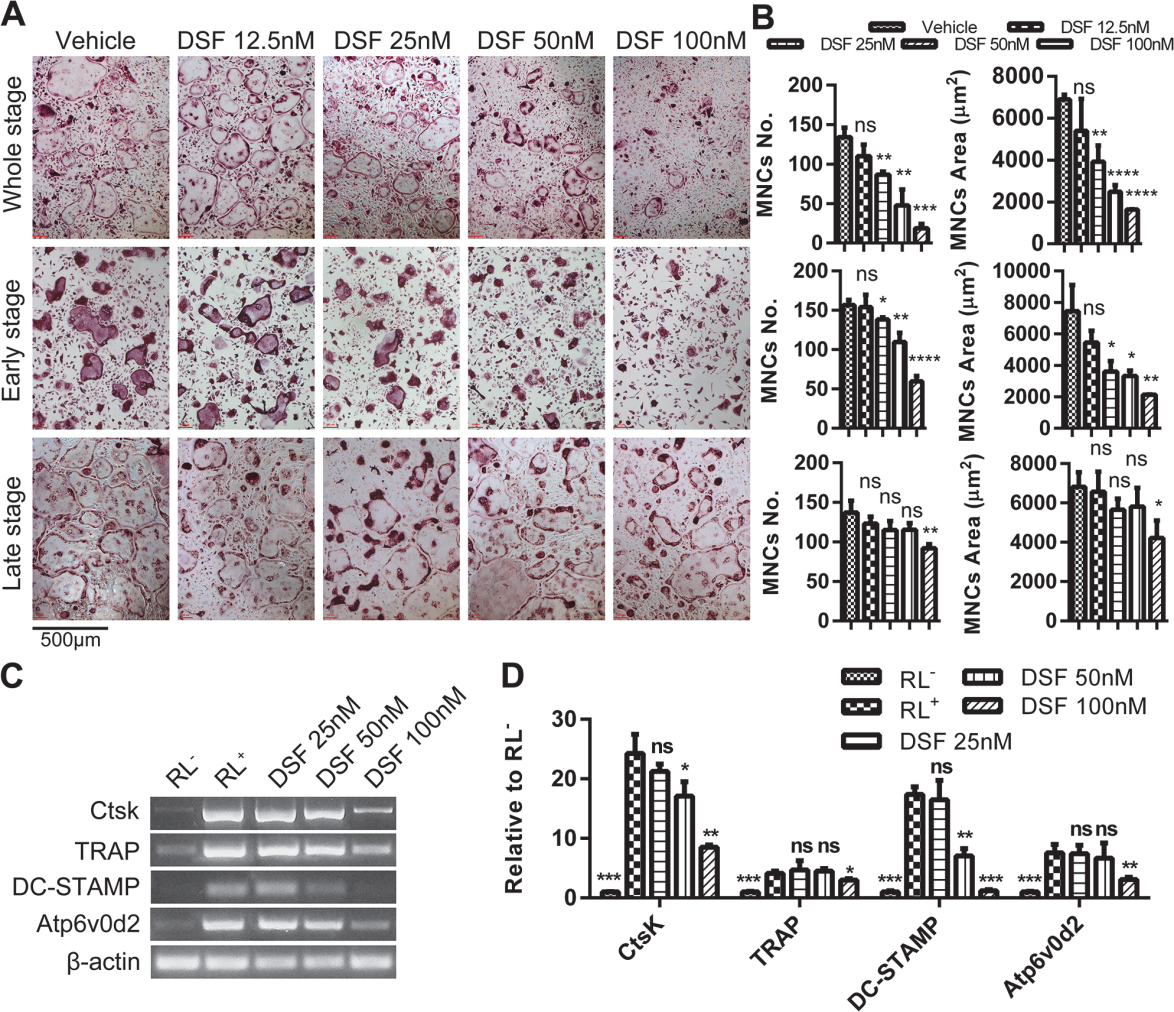
DSF inhibited osteoclastogenesis during the early stage of differentiation *in vitro*. (A) Time and dose-dependent inhibitory effect of DSF on OC formation. During a typical 5-day culture of osteoclastogenesis, DSF treatment on days 1–2 are defined as the early stage and treatment on days 3–4 as the late stage. Freshly isolated M-CSF-dependent BMMs cultured in the presence of rRANKL (100ng/ml) and various concentrations of DSF (12.5 nM, 25nM, 50 nM and 100 nM) for 4 days were fixed with 4% PFA followed by TRAP staining for the visualization of multinucleated OCs (scale bar = 500μm). (B) The number and average size in areas (mm^2^) of TRAP-positive multinucleated OCs (≥3 nuclei) at the different time points were quantified (mean ± SD; *: p<0.05, **: p<0.01, ***: p<0.001, ****: p<0.0001, ns: not significant against vehicle). (C) Effect of DSF on OC-specific gene expression. Total RNA was isolated from BMM cells cultured in the presence or absence of rRANKL and various doses of DSF (25, 50 and 100nM) for 4 days. cDNA was synthesized using 1μg of total RNA and subjected to PCR amplification using specific primers for OC-specific genes, CtsK, TRAP, DC-STAMP, Atp6v0d2 and housekeeping gene β-actin. PCR products were separated and analysed on 1.5% agarose gels. (D) The relative levels of gene expression was calculated as ratios against β-actin (mean ± SD; *: p<0.05, **: p<0.01, ***: p<0.001, ns: not significant against RL^+^).

To investigate the effect(s) of DSF directly on OC formation, we next cultured mouse BMMs in the presence of pro-osteoclastogenic media (*i*.*e*. M-CSF (30 ng/ml) and rRANKL (100 ng/ml)) in either the presence of DSF at varying concentrations (12.5 nM, 25 nM, 50 nM and 100 nM) or vehicle control for various time points up to 4-days upon which TRAP-positive (pink reaction product) multinucleated OCs (≥3 nuclei) typically form under control conditions (Fig 2A and 2B). As shown in Fig 2A and 2B, DSF dose-dependently reduced both the number and size/area of TRAP-positive OCs. This reduction in OC formation was primarily observed when DSF was administered to cultures during the early stage (Day 1–2), but not late stage (Day 3–4) of the differentiation process (except for 100 nM DSF which reached significance at both time points) (Fig 2B). This indicates that the inhibitory action of DSF predominates during the early stage of RANKL-driven OC differentiation.

To further validate the impact of DSF on OC formation, we have examined how DSF will affect human OC differentiation. Monocytes were isolated from whole blood of two healthy participants and cultured to mature OCs according to established protocols [25]. Varying concentrations of DSF (12.5 nM, 25 nM, 50 nM and 100 nM) has been administered during the whole culture period. As shown in S1A Fig and S1B Fig, DSF exhibited similar dose-dependent inhibitory effect on human OC formation as observed in mouse osteoclastogenesis.

**Fig 3:**
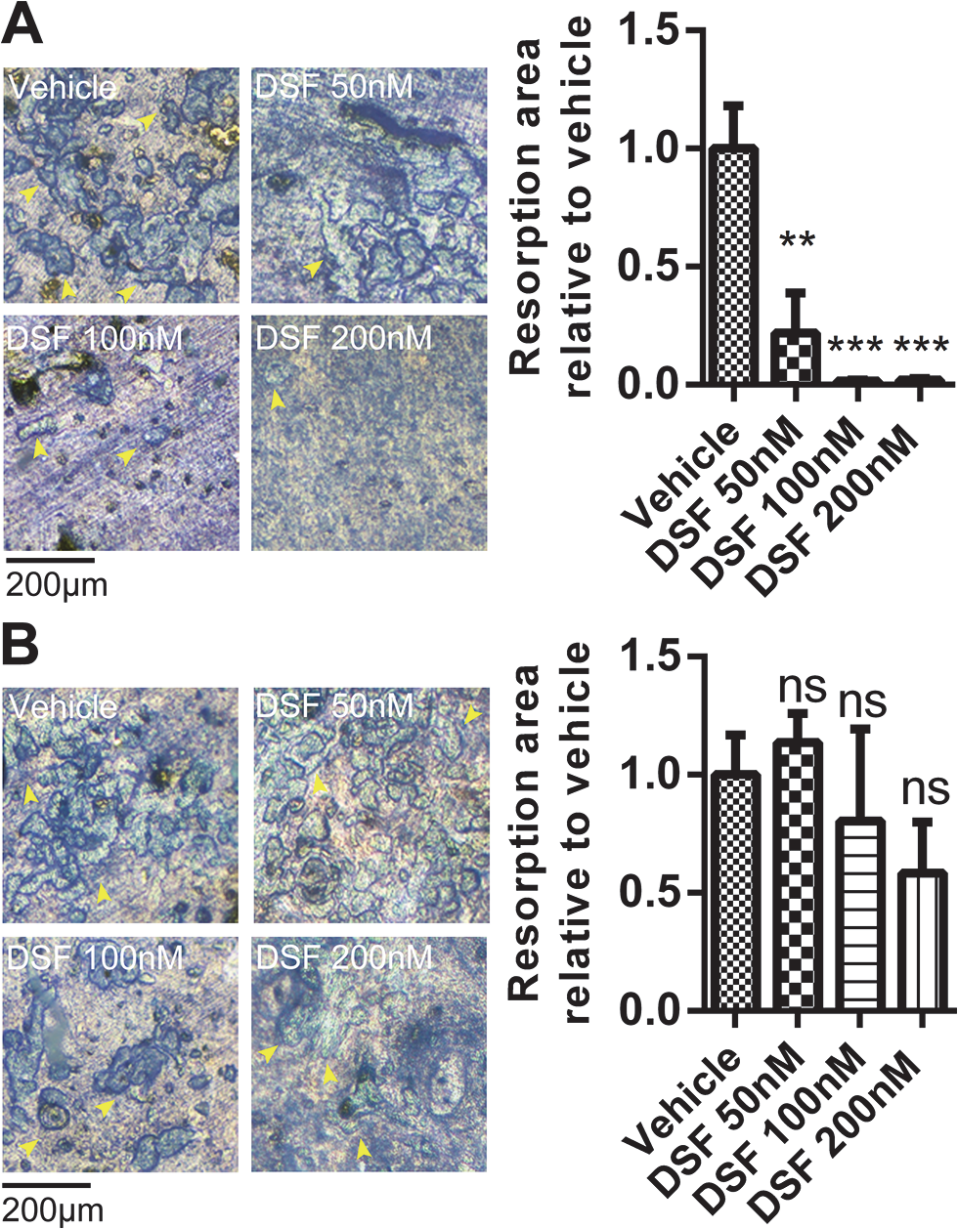
Effect of DSF on bone resorption. (A) BMMs were seeded on bone discs and cultured to form OCs in the presence or absence of DSF (50 nM, 100 nM and 200 nM). (B) BMMs were cultured to OCs on bone discs and then treated with DSF (50 nM, 100 nM and 200 nM) for 48 hours. Bone discs were fixed and assessed for bone resorption by toluidine blue staining (resorption pits shown by yellow arrow heads). The total resorbed area on each bone disc was quantified as a percentage of the total bone disc area (mean ± SD; **: P<0.01, ***: P<0.001, ns: not significant against vehicle).

Consistent with the impairment of osteoclastogenesis, the expression levels of several established OC marker genes, including cathepsin K (CtsK), tartrate resistant acid phosphatase (TRAP), dendritic cell-specific transmembrane protein (DC-STAMP) and Atp6v0d2, were also notably reduced upon exposure to DSF, which was most evident at 100 nM DSF (Fig 2C and 2D). On the other hand, the bone resorption activity of OCs was significantly reduced when DSF was administered throughout the differentiation of BMMs into OCs cultured on bone (up to 7 days) (Fig 3A). However, this effect was minor when DSF was administered for 48 hours to pre-formed mature OCs on bone (Fig 3B). Taken together, these data indicate that DSF impairs OC differentiation but does not influence OC bone resorptive function.

### Disulfiram attenuates RANKL-induced MAPK and NF-κB signaling in BMMs

**Fig 4:**
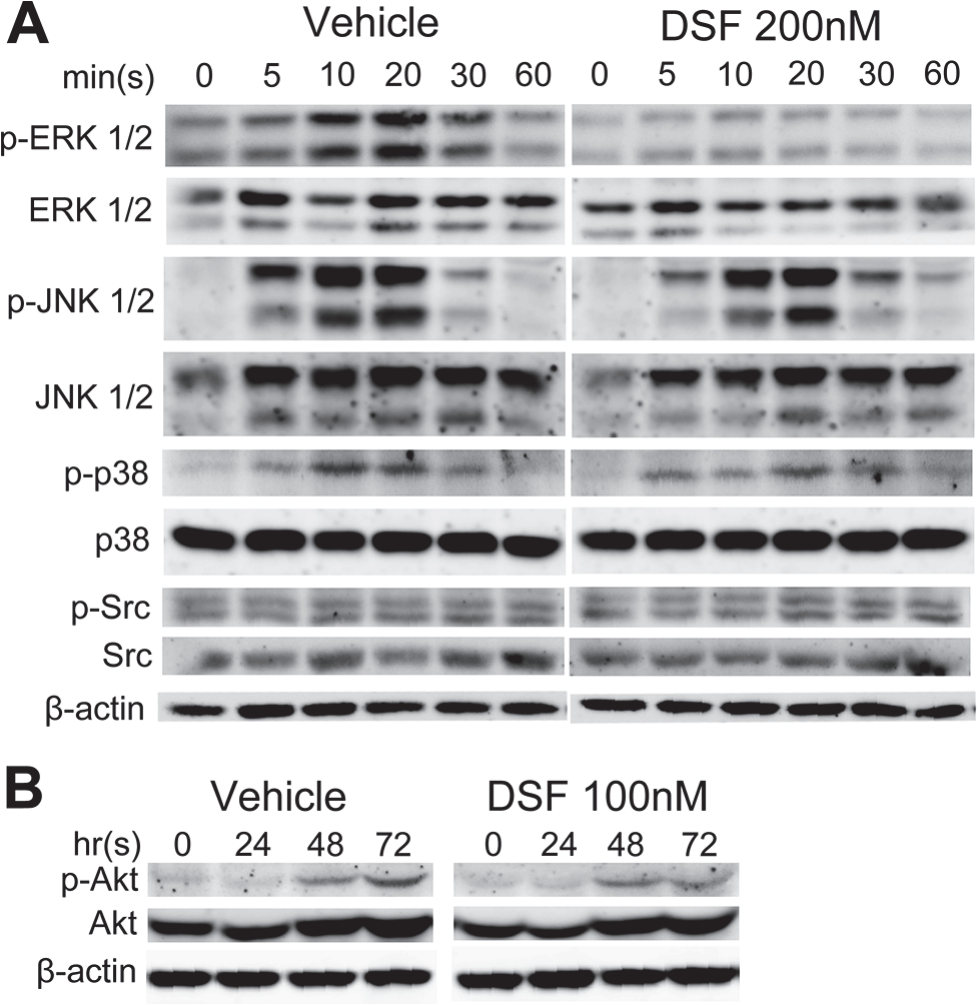
DSF attenuates RANKL-induced MAPK signaling in BMMs. Total cell lysates were extracted from BMMs treated with rRANKL for 0, 5, 10, 20, 30 and 60 mins (A) or for 0, 24, 48 and 72 hrs (B) in the presence or absence of DSF (200nM or 100nM). Proteins were separated on 12.5% SDS-PAGE gel, transferred onto nitrocellulose membranes, and immunoblotted sequentially with antibodies to different components of the MAPK and SAPK signaling pathways (ERK, JNK, p38, Src and Akt). β-actin was used as internal loading control. Results shown represent one of three independent experiments.

To establish a mechanistic basis for the observed decrease in OC differentiation during the early stage of osteoclastogenesis, we next examined the effect of DSF on key RANKL-mediated signaling cascades including MAPK and stress-activated protein kinase (SAPK) phosphorylation. As expected, addition of rRANKL induced robust ERK1/2 phosphorylation with peak phosphorylation observed after 10–20 min post-stimulation. By comparison, ERK1/2 phosphorylation was markedly attenuated following brief exposure (1hr) to high DSF concentration (200 nM). On the other hand, DSF did not influence the phosphorylation signatures of several additional key signaling MAPK/SAPK proteins examined including p38, JNK 1/2, Src and Akt (Fig 4A and 4B).

**Fig 5:**
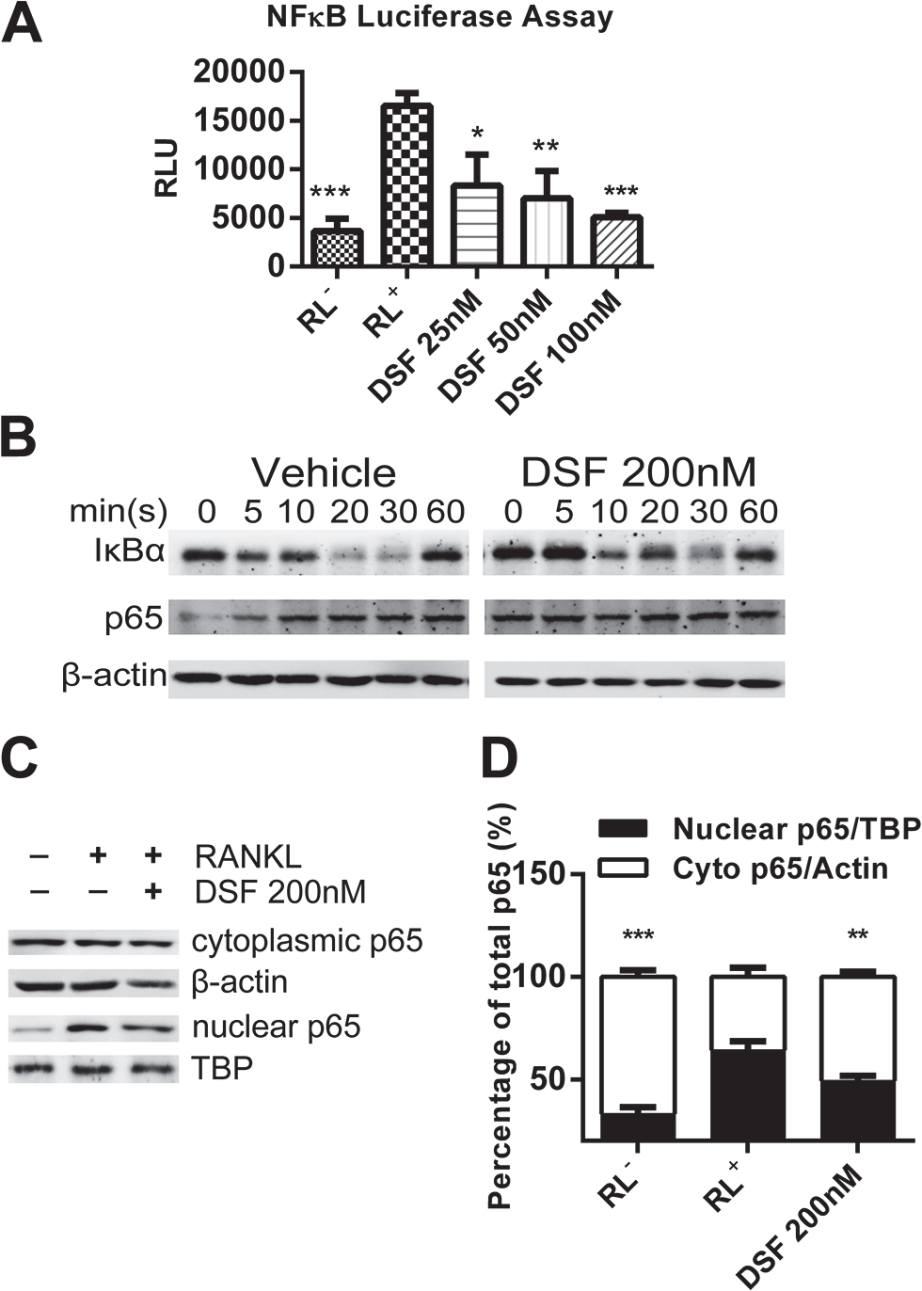
DSF suppresses RANKL-induced activation of NF-κB. (A) DSF suppressed RANKL-induced NF-B luciferase activity. RAW264.7 cells stably expressing a NF-B-driven luciferase reporter construct were pre-treated with varying doses of DSF for 1 hr followed by rRANKL stimulation. Luciferase activity was assessed after 8 hrs of RANKL stimulation (mean ± SD; *: P<0.05, **: P<0.01, ***: P<0.001 against RL^+^). (B) DSF delayed IκBα degradation. Total cell lysates were extracted from BMMs treated with rRANKL for 0, 5, 10, 20, 30 and 60 mins in the presence or absence of DSF (200 nM). Proteins were separated on 12.5% SDS-PAGE gel, transferred onto nitrocellulose membranes, and immunoblotted sequentially with antibodies to IκBα and p65. β-actin was used as internal loading control. (C and D) DSF attenuated the nuclear translocation of p65. BMMs were treated with RANKL for 1 hr in the presence or absence of DSF (200 nM). Cytoplasmic and nuclear fractions were extracted using NE-PER Nuclear and Cytoplasmic Extraction Reagents, separated on 12.5% SDS-PAGE gel, and then transferred onto nitrocellulose membrane. Membrane was immunoblotted for p65. -actin and TBP were used as loading controls for cytoplasmic and nuclear fractions respectively. Results shown represent one of three independent experiments.

Next, we assessed the effects of DSF on NF-κB signaling pathways. For this we monitored the effects of DSF on NF-κB transcriptional activity using mouse monocytic pre-OC cell line RAW264.7 cells that were stably transfected with an NF-κB-driven luciferase reporter construct [26]. As expected, RANKL treatment alone elicited an increase in NF-κB promoter driven luciferase gene expression (~5-fold) as compared to cells cultured in medium alone (i.e. without rRANKL, negative control) (Fig 5A). By comparison, treatment of RAW264.7 cells with DSF (25 nM, 50 nM and 100 nM) significantly impaired RANKL-induced NF-κB transcriptional activation. In keeping with these observations, DSF treatment (200 nM) reduced the RANKL-induced degradation and nuclear translocation of core NF-κB components including IκBα and p65 (Fig 5B and 5C). Whereas BMMs treated with RANKL alone showed maximal degradation of IκBα at 20–30 mins, incubation with DSF postponed peak IκBα degradation to 30 mins post-RANKL stimulation (Fig 5B). Similarly, while the total cellular protein level of p65 remained unchanged in BMMs stimulated with RANKL in either the presence or absence of DSF (200 nM) for 60 mins, cytoplasmic and nuclear protein partitioning revealed a reduced ratio of p65 cytosol:nuclear translocation in the presence of DSF (Fig 5C and 5D) relative to the β-actin and TATA binding protein (TBP), loading controls for cytosolic and nuclear proteins, respectively. Collectively, these data indicate that DSF directly attenuates RANKL-induced NF-κB and ERK signaling cascades during OC differentiation.

### Disulfiram impairs NFATc1 transactivation in RANKL-stimulated BMMs

**Fig 6:**
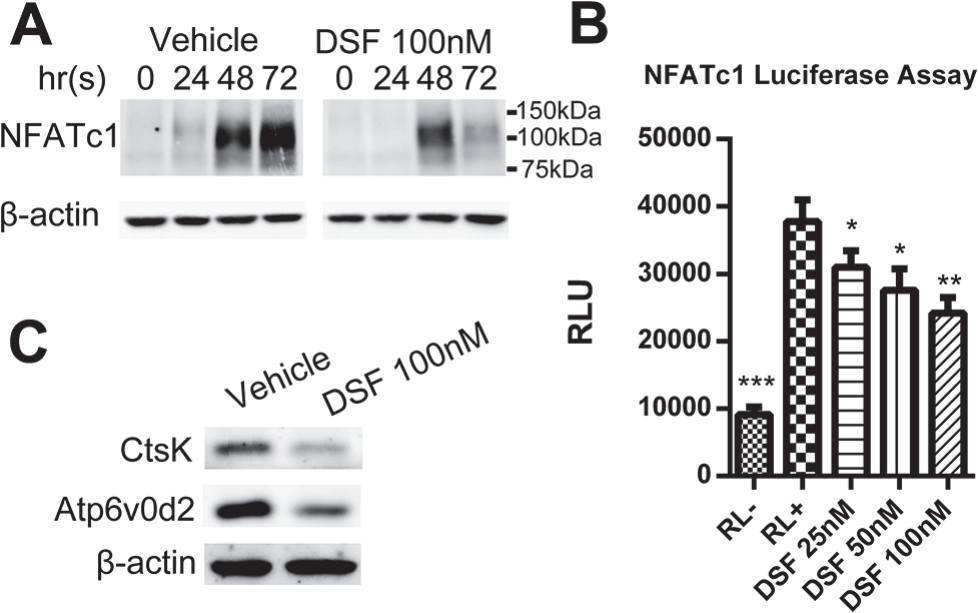
DSF inhibited RANKL-induced NFATc1 signaling. (A) DSF attenuated RANKL-induced NFATc1 protein expression. Total cell lysates were extracted from BMMs treated with rRANKL for 0, 24, 48 and 72 hrs in the presence or absence of DSF (100nM). Proteins were separated on 10% SDS-PAGE gel, transferred onto nitrocellulose membranes, and immunoblotted with specific antibody to NFATc1. β-actin was used as internal loading control. (B) DSF dose-dependently inhibited RANKL-induced NFATc1 luciferase activity. RAW264.7 cells stably expressing a NFATc1-driven luciferase reporter construct was pre-treated with varying doses of DSF (25, 50, 100nM) for 1 hr followed by rRANKL stimulation. Luciferase activity was assessed after 24 hrs of RANKL stimulation (mean ± SD; *P<0.05, **P<0.01, ***P<0.001 against RL^+^). (C) DSF impaired NFATc1 downstream effector proteins CtsK and Atp6v0d2 expression. Total cell lysates were extracted from BMM-derived OCs cultured in the presence or absence of DSF (100nM). Proteins were separated on 12.5% SDS-PAGE gel, transferred onto nitrocellulose membranes, and immunoblotted with specific antibodies to CtsK and Atp6v0d2. -actin was used as internal loading control. Results shown represent one of three independent experiments.

Aforementioned, together with NF-κB, activation of NFATc1 is indispensable for OC differentiation [30]. Therefore, we further probed for the expression of NFATc1 in RANKL stimulated BMMs in the presence or absence of DSF (100 nM) by immunoblotting. As expected, RANKL alone induced a prominent up-regulation of NFATc1 expression 48–72 hrs post-incubation (Fig 6A). By comparison, DSF treatment markedly diminished RANKL-induced NFATc1 expression levels at 72 hrs. Consistently, NFATc1 transactivation, as monitored by RAW264.7 cells stably transfected with an NFATc1-driven luciferase reporter construct, was significantly impaired across all concentrations of DSF tested (25 nM, 50 nM and 100 nM) as compared to the control in response to RANKL-stimulation (Fig 6B). This reduction in NFATc1 transactivation corresponded with a notable reduction in the protein expression levels of several downstream osteoclastic markers known to be transcriptionally regulated by NFATc1 including CtsK and Atp6v0d2 (Fig 6C). Together, the data extend NFATc1 to the list of key signaling molecules modulated by DSF during OC differentiation.

### Disulfiram does not inhibit V-ATPase mediated acidification in osteoclasts

**Fig 7:**
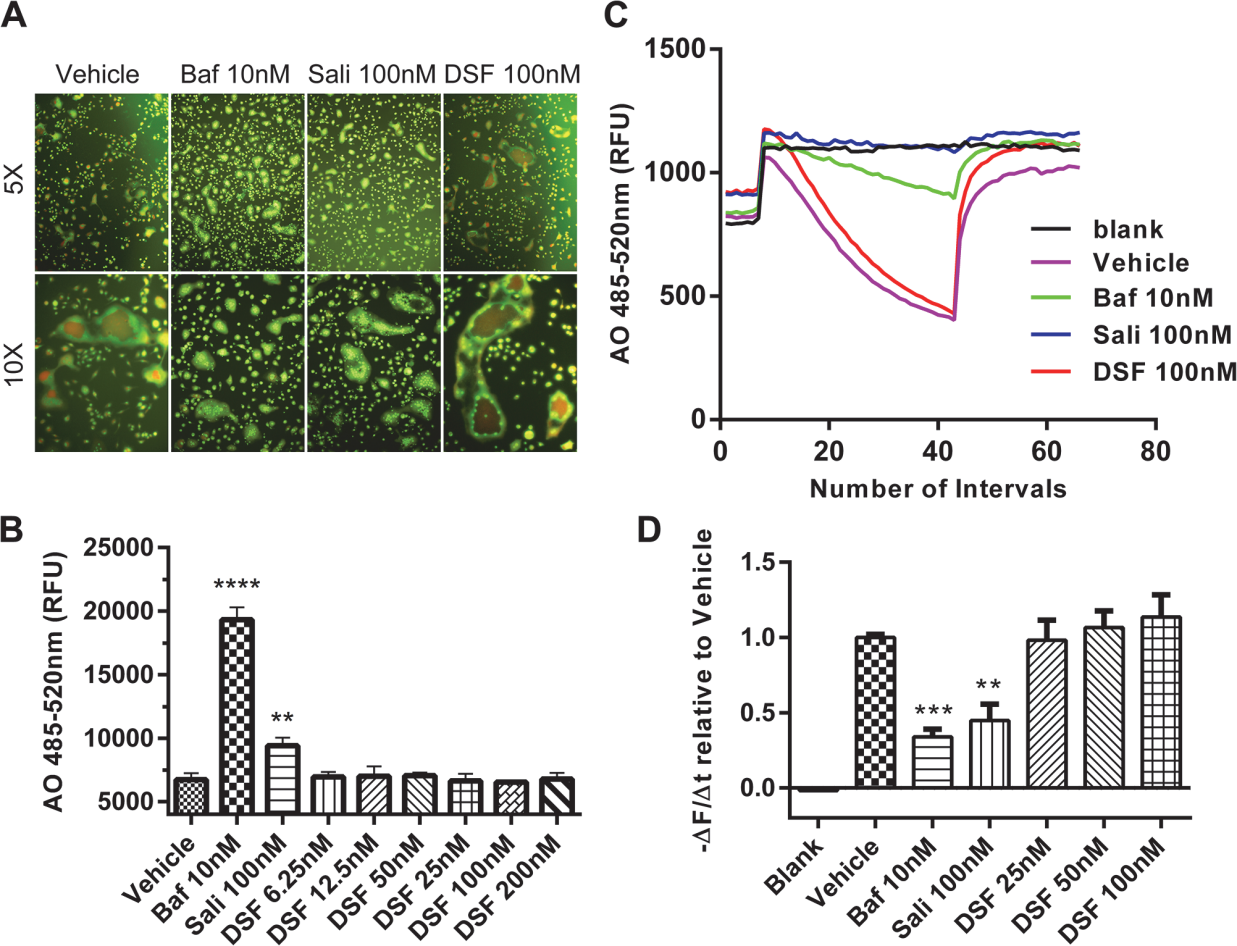
V-ATPase proton pump is not the target of DSF. (A) DSF does not affect V-ATPase-mediated acidification in OCs. Image merge of green and red fluorescence spectra of acridine orange (AO) fluorescence quenching from BMM-derived OCs pre-treated for 12 hrs with various concentrations of DSF (6.25, 12.5, 25, 50, 100 and 200 nM), Baf (10 nM) or SaliPhe (100 nM) followed by incubation with 5 μg/ml AO for 15 mins at 37°C. Cells were first excited with wavelength of 485 nm. Fluorescence shift of AO from green to red indicate normal intracellular acidification. (B) Fluorescence intensity at excitation 485 nm and emission 535 nm was quantified using a spectrophotometer (mean ± SD; **: P<0.01, ****: P<0.0001 against vehicle). (C and D) DSF does not affect V-ATPase-mediated proton transport in isolated microsomes. Microsomes isolated from RAW264.7 cells was subjected to AO proton transport assay in the presence or absence of DSF (100 nM), Baf (10 nM) or SaliPhe (100 nM). Proton transport was initiated by the addition of ATP and influx was detected by the fluorescence intensity measured with excitation 485 nm and emission 535 nm and results are represented as the initial rate of acidification (-∆F/∆t as calculated from the slope generated by the first 60 seconds after ATP supplement and normalized to vehicle). Results shown represent one of three independent experiments.

Finally, given that DSF has been recently reported to inhibit V-ATPase-mediated acidification in eukaryotes [21] together with the vital importance of this proton pump in OC differentiation and function [11, 31, 32], we tested whether DSF modulates acidification in OCs. To this end, we monitored the effect of DSF on intracellular acidification in BMM-derived OCs by acridine orange (AO) fluorescence quenching assay [33]. AO fluorescence quenching assays are routinely employed to monitor changes in the intracellular acidification status of a variety of cells and organelles [34]. Upon entering acidified compartments, unprotonated AO (green light; Em 535 nm) becomes protonated and emits orange/red light (Em 580 nm) when excited by blue light (Ex 492 nm). AO fluorescence intensity can then be measured by spectrophotometry as a direct readout of acidification levels. As shown in Fig 7A, whereas established V-ATPase inhibitors/macrolides Baf (10 nM) and SaliPhe (100 nM) [34, 35] potently blocked AO quenching and thus intracellular acidification in OCs as signified by the predominantly green fluorescence signal emitted by the Ex485/Em535 fluorescence spectra, DSF (100 nM) failed to elicit a fluorescence shift of AO fluorescence away from typical acidified orange/red color (as observed in vehicle-treated control) to green suggesting that DSF did not influence intracellular acidification. Quantification of AO fluorescence intensity at Ex485/Em535 using a spectrophotometer further confirmed the dose-dependent elevation of intracellular pH levels (decreased acidity) by Baf and SaliPhe while DSF treated shown no quantifiable shift away from control acidification levels (Fig 7B). Additionally, AO staining was adopted to validate whether DSF affects human OC V-ATPase activities. As shown in S1C Fig, untreated human OCs exhibited bright orange staining on the site of podosomal belt which is a actin-rich structure on the periphery of a non-resorbing osteoclast [36], while Baf treatment potently impaired the acidification by inhibiting V-ATPaseS. Consistent with previous results, DSF failed to exert obvious inhibitory effects on human OC V-ATPases.

We also verified these findings biochemically using a microsome-based proton influx assay to directly monitor the effects of DSF in relation to bafilomycin and SaliPhe on V-ATPase mediated H^+^ transport. Acidic influx was initiated by the addition of ATP as indicated by the steady decline in fluorescence intensity slope over time (Fig 7C). As expected, both bafilomycin (10 nM) and SaliPhe (100 nM) significantly inhibited transmembrane proton transport as indicated by the unchanged fluorescence intensity level over time following ATP addition. By comparison, DSF (25-100nM) failed to block H^+^ transport (Fig 7C and 7D). The slope (-∆F/∆t) of each curve was calculated and demonstrated as percentage of vehicle (Fig 7D). Taken together, these data indicate that DSF does not inhibit the OC V-ATPase complex.

## Discussion

Disulfiram has historically been employed for the treatment of chronic alcoholism [37]. The major findings of the present study indicate that DSF treatment dose-dependently impairs OC formation *in vitro* at the early stages of differentiation. Moreover, this inhibition in OC differentiation appears to be due, largely in part, to the suppression of several key RANKL-induced signaling cascades including ERK, NF-κB and NFATc1. Thus, our data demonstrate, for the first time, that DSF also possesses antiresorptive properties.

By reevaluating the cellular and mechanistic actions of DSF, our study highlights the potential of using pre-existing market approved agents as alternative anti-bone resorption therapies. In support of this, previous studies by Pilar Peris *et al*. [38] reported that DSF treatment, in combination with alcohol abstinence (for up to 2 yrs), correlated with an increase in lumbar and femoral BMD in alcoholics. While at this point in time we cannot rule out the possibility that the observed changes in BMD of these chronic alcoholics were not simply a culmination of factors influenced by alcohol intake (*i*.*e*., dietary, hormone levels, physical activities *etc)*, it is tempting to speculate that our *in vitro* findings may provide a cellular basis for this BMD restoration i.e. through the direct actions of DSF on OC formation. Moreover, a more recent study by Monika Mittal *et al*. [39] indicated the DSF suppressed osteoblast survival and differentiation via inhibition of Aldh2 function, which in turn suggests that DSF might display biphasic effect on bone remodeling cycle and have greater effects towards preserving current bone mass. Therefore, reexamination of DSF on a non-alcoholic cohort of osteoporotic patients will be required to substantiate this position.

By combining immunoanalytical methods with transcriptional reporter assays we demonstrate that a short incubation with DSF inhibited the nuclear translocation of p65 after stimulation with RANKL and the effect was mediated by the reduced degradation of IκBα, the inhibitory subunit of NF-κB. Since IκBα has been suggested to be degraded in the proteasome and DSF has been shown to possess proteasome inhibition potential, we speculate that the general blockade of DSF on NF-κB transactivation is through proteasome inhibition [40–42].The corresponding decrease in OC formation and reduction in NF-κB signaling is consistent with the notion that NF-κB activation is vital to osteoclastogenesis which is best exemplified in studies of p50/p52 double knockout of NF-κB machinery in mice which exhibit greatly suppressed OC formation and osteopetrosis [43]. The inhibitory actions of DSF on NF-κB signaling also parallel the findings of other established NF-κB blockers such as the IκB supersupressor, which reported a similar reduction in OC differentiation and activation *in vitro* [44].

Along with NF-κB, we found that DSF impaired the transactivation of NFATc1 and expression of downstream transcriptional targets including DC-STAMP and Atp6v0d2, each essential for OC formation. Given the crucial role of NFATc1 regulation in the early phases of OC formation, it is likely that this target accounts, at least in part, for the observed inhibition of osteoclastogenesis following continuous and/or early stage DSF treatment. On the other hand, when administered at late-stages of differentiation, DSF had little influence of osteoclastogenesis probably because NFATc1 expression had already elicited the induction of the necessary transcriptional machinery required for OC precursor commitment and fusion [12, 45].

In the present study we found that DSF treatment inhibited ERK1/2 phosphorylation but did not influence other key pathway components p38, JNK, Src and Akt. These findings were not entirely unexpected as DSF has been previously shown to modulate MAPK/SAPK signaling in various cancer cell lines when employed in combination with CuCl_2_ [18,35].

Along with the abovementioned core RANKL-mediated signaling machinery, the V-ATPase proton pump plays an essential role in OC formation and function. During bone resorption V-ATPases are clustered at high density on the ruffled border to facilitate extracellular acidification and bone resorption, thus the V-ATPase complex has long been considered a target for antiresorptive therapy [46]. Interestingly, DSF has recently been shown to be an inhibitor of the yeast V-ATPase proton pump. However, in our hands, using multiple lines of cellular and biochemical investigation, we failed to detect any inhibitory effects of DSF on V-ATPase-mediated acidification in OCs. Whereas canonical V-ATPase inhibitors Baf and SaliPhe exhibited potent inhibitory effects on acidification, as monitored by AO fluorescence quenching and proton influx assays, DSF showed no obvious inhibitory effects across all doses examined (up to 200 nM). While the precise reason for this discrepancy warrants further study, it may simply reflect differences in the pharmacological action(s) of DSF and/or the configuration of the V-ATPase complex observed between yeast and mammalian cells [32].

Finally, although our findings provide new mechanistic insights for DSF as a candidate agent resorptive agent, several limitations remain to be addressed. After successfully evaluating the known RANKL-induced signaling cascades, we found that with the exception of ERK 1/2, DSF did not influence the phosphorylation signatures of other major MAPK/SAPK signaling proteins including p38, JNK 1/2, Src and Akt. However the difference in ERK phosphorylation was not addressed mechanistically. Furthermore, *in vivo* models of osteoporosis (such as OVX-induced bone loss in mice) along with future clinical trials will be the focus of further studies in order to strengthen the resolve of our *in vitro* findings and potentially elevate DSF to the list of established antiresorptive agents.

## Conclusions

In summary, we demonstrate, for the first time, that DSF attenuates both mouse and human osteoclastogenesis *in vitro* by disruption of several key RANKL-induced signaling pathways. We posit that DSF may therefore be a potential candidate treatment for osteoclast-mediated bone diseases like osteoporosis.

## Supporting Information

S1 Fig The effect of DSF on human osteoclasts. (A) Dose-dependent inhibitory effect of DSF on OC formation. Freshly isolated human monocytes were cultured in the presence of rRANKL (100ng/ml) and human M-CSF in the presence or absence of various concentrations of DSF (12.5 nM, 25nM, 50 nM and 100 nM) for 10 days and fixed with 4% PFA followed by TRAP staining for the visualization of multinucleated OCs (scale bar = 500μm). (B) The number and average size in areas (mm^2^) of TRAP-positive multinucleated OCs (≥3 nuclei) were quantified (mean ± SD; *: p<0.05, **: p<0.01, ***: p<0.001, ****: p<0.0001, ns: not significant against vehicle). (C) Image merge of green and red fluorescence spectra of AO fluorescence quenching from human OCs. Cells were pre-treated for 12 hrs with DSF (100 nM) or Baf (10 nM) followed by incubation with 5 μg/ml AO for 15 mins at 37°C (scale bar = 100μm). Results shown represent one of two independent experiments.
(TIF)
