# Supplementary figures and images for: Disulfiram Attenuates Osteoclast Differentiation In Vitro: A Potential Antiresorptive Agent
Source: PLoS One. 2015 Apr 30;10(4):e0125696. doi: 10.1371/journal.pone.0125696 (PMC4416043; doi:10.1371/journal.pone.0125696)

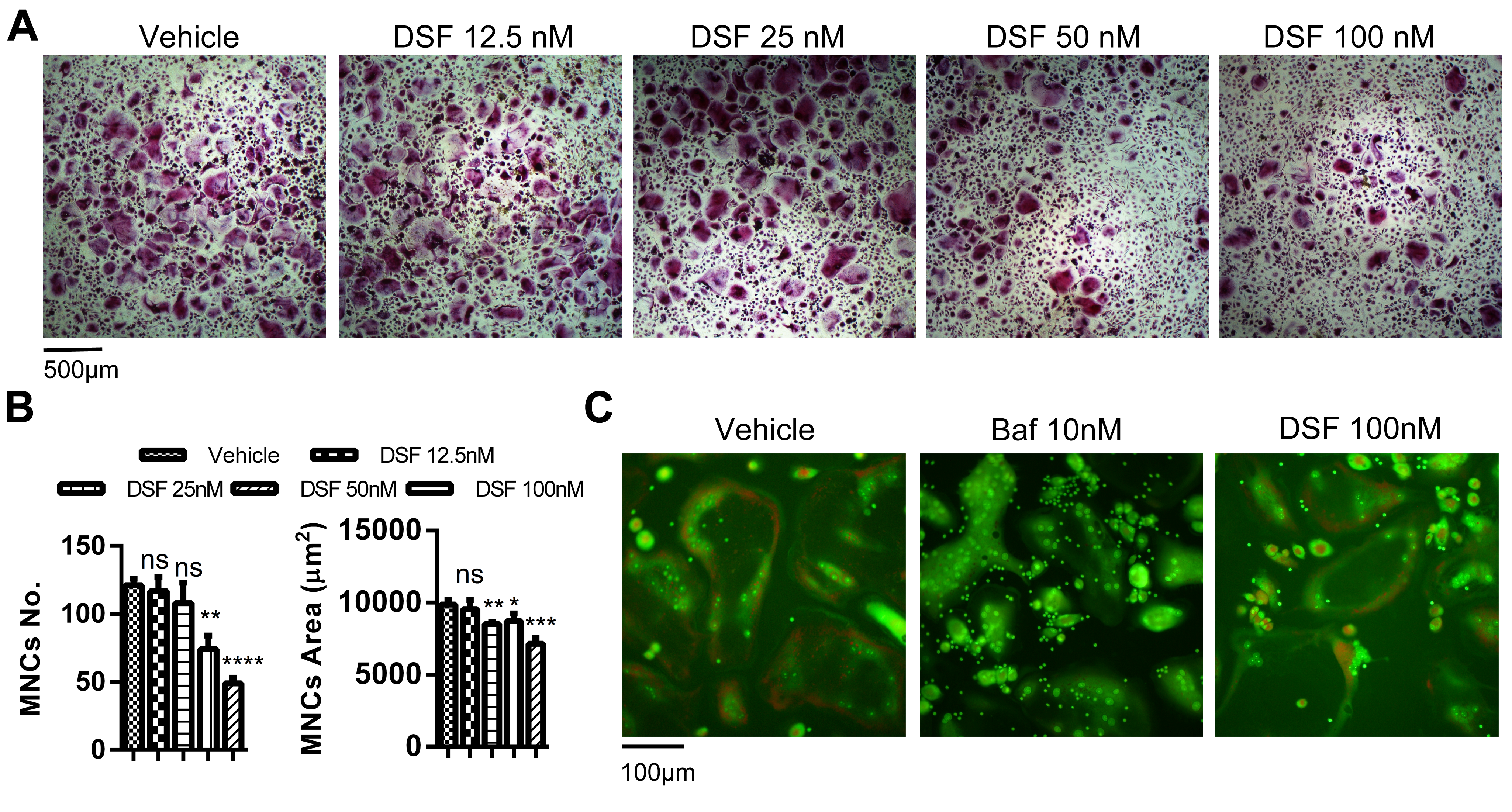

Supplement: S1 Fig — (A) Dose-dependent inhibitory effect of DSF on OC formation. Freshly isolated human monocytes were cultured in the presence of rRANKL (100ng/ml) and human M-CSF in the presence or absence of various concentrations of DSF (12.5 nM, 25nM, 50 nM and 100 nM) for 10 days and fixed with 4% PFA followed by TRAP staining for the visualization of multinucleated OCs (scale bar = 500μm). (B) The number and average size in areas (mm2) of TRAP-positive multinucleated OCs (≥3 nuclei) were quantified (mean ± SD; *: p<0.05, **: p<0.01, ***: p<0.001, ****: p<0.0001, ns: not significant against vehicle). (C) Image merge of green and red fluorescence spectra of AO fluorescence quenching from human OCs. Cells were pre-treated for 12 hrs with DSF (100 nM) or Baf (10 nM) followed by incubation with 5 μg/ml AO for 15 mins at 37°C (scale bar = 100μm). Results shown represent one of two independent experiments. (TIF) [file pone.0125696.s001.tif]
